# Integrated oral microgel system ameliorates renal fibrosis by hitchhiking co-delivery and targeted gut flora modulation

**DOI:** 10.1186/s12951-024-02586-2

**Published:** 2024-06-01

**Authors:** Yu Hou, Lin Zhu, Xiaofeng Ye, Qiaoying Ke, Qibin Zhang, Xiaowei Xie, Ji-gang Piao, Yinghui Wei

**Affiliations:** School of Pharmaceutical Sciences, Zhejiang Chinese Medical University, Hangzhou, 311402 China

**Keywords:** Nanoassembly, Emodin, Asiatic acid, Probiotics, Microgels, Synergistic treatment and moderation, Renal fibrosis

## Abstract

**Background:**

Renal fibrosis is a progressive process associated with chronic kidney disease (CKD), contributing to impaired kidney function. Active constituents in traditional Chinese herbs, such as emodin (EMO) and asiatic acid (AA), exhibit potent anti-fibrotic properties. However, the oral administration of EMO and AA results in low bioavailability and limited kidney accumulation. Additionally, while oral probiotics have been accepted for CKD treatment through gut microbiota modulation, a significant challenge lies in ensuring their viability upon administration. Therefore, our study aims to address both renal fibrosis and gut microbiota imbalance through innovative co-delivery strategies.

**Results:**

In this study, we developed yeast cell wall particles (YCWPs) encapsulating EMO and AA self-assembled nanoparticles (NPYs) and embedded them, along with *Lactobacillus casei* Zhang, in chitosan/sodium alginate (CS/SA) microgels. The developed microgels showed significant controlled release properties for the loaded NPYs and prolonged the retention time of *Lactobacillus casei* Zhang (*L. casei* Zhang) in the intestine. Furthermore, in vivo biodistribution showed that the microgel-carried NPYs significantly accumulated in the obstructed kidneys of rats, thereby substantially increasing the accumulation of EMO and AA in the impaired kidneys. More importantly, through hitchhiking delivery based on yeast cell wall and positive modulation of gut microbiota, our microgels with this synergistic strategy of therapeutic and modulatory interactions could regulate the TGF-β/Smad signaling pathway and thus effectively ameliorate renal fibrosis in unilateral ureteral obstruction (UUO) rats.

**Conclusion:**

In conclusion, our work provides a new strategy for the treatment of renal fibrosis based on hitchhiking co-delivery of nanodrugs and probiotics to achieve synergistic effects of disease treatment and targeted gut flora modulation.

**Graphical Abstract:**

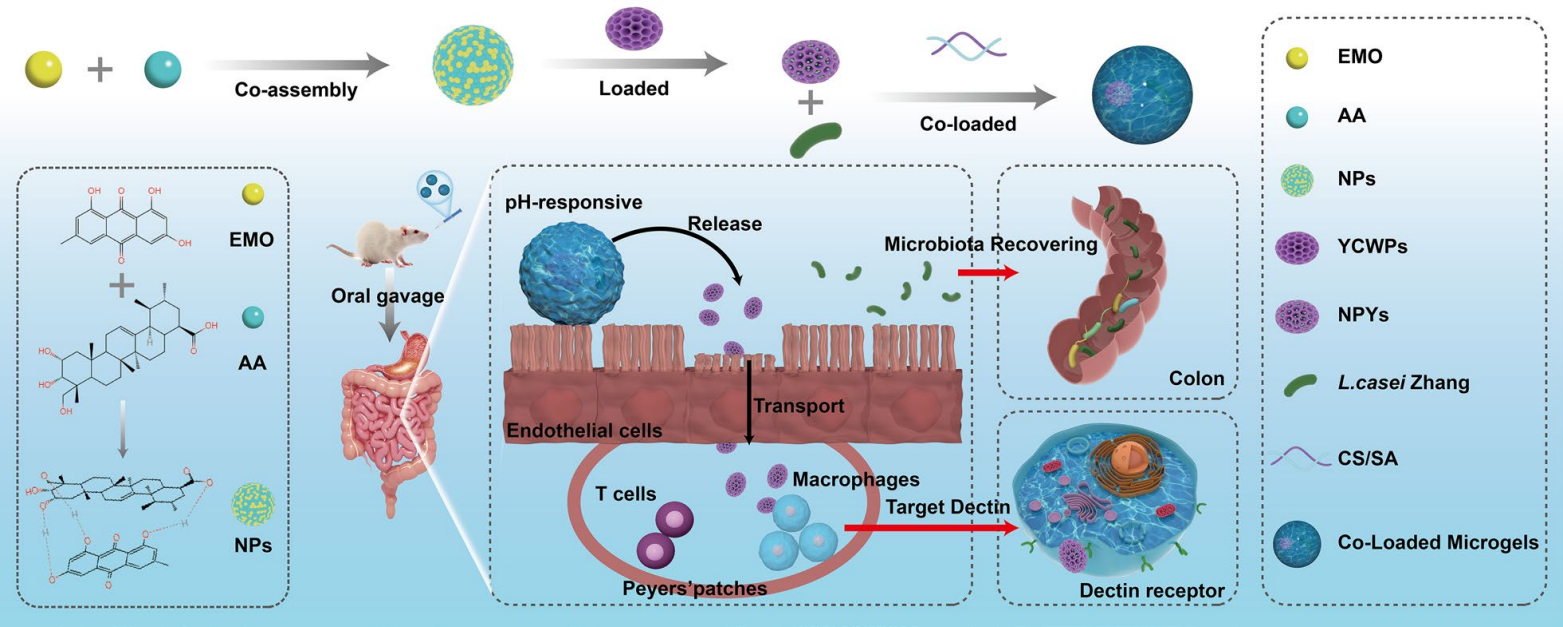

**Supplementary Information:**

The online version contains supplementary material available at 10.1186/s12951-024-02586-2.

## Introduction

Chronic kidney disease (CKD) represents a significant health challenge worldwide, marked by the insidious progression of renal fibrosis [1]. This pathological process underpins the development of glomerulosclerosis and renal interstitial fibrosis, hallmarks of CKD [2]. Central to renal fibrosis are two key features: glomerular sclerosis and renal interstitial fibrosis [3]. Remarkably, recent research has unveiled a compelling connection between renal fibrosis and disruptions in gut microbiota composition [4–6]. This discovery highlights the intricate relationship between the gut and the kidneys, wherein renal fibrosis contributes to gut dysbiosis, leading to a decrease in short-chain fatty acids (SCFAs) and an increase in the production of uremic nephrotoxins [7–9]. Uremic toxins, in turn, exacerbate intracellular oxidative stress, further intensifying kidney tubule interstitial fibrosis and hindering toxin excretion, therefore creating a self-reinforcing feedback loop [10–12]. Despite the existing clinical treatments for renal fibrosis, such as angiotensin inhibitors and transforming growth factor-beta (TGF-β) inhibitors [13], they exhibit limitations like dose-dependent effects, extrarenal impacts, and aldosterone escape, resulting in suboptimal outcomes [14]. Therefore, novel strategies are urgently needed to simultaneously address renal fibrosis and the imbalance in intestinal flora.

Many studies have attributed a key role in fibrotic progression to TGF-β [15, 16], which executes its biological functions through downstream activation of the recombinant mothers against decapentaplegic homolog (Smad) signaling pathway [17]. TGF-β1, the most abundant isoform among TGF-β family members, can be secreted by various renal cells and infiltrated by inflammatory cells [18, 19]. These inflammatory cells constantly infiltrate the kidney, producing pro-inflammatory cytokines that induce kidney inflammation [20, 21]. These aforementioned observations suggest the critical involvement of the TGF-β/Smad pathway and inflammatory proteins in the development of renal fibrosis [22, 23]. Emodin (EMO), derived primarily from the roots and stems of *Rhubarb* and *Polygonum cuspidatum* [24, 25], has demonstrated potent anti-fibrotic properties. Research indicates that EMO can inhibit epithelial-mesenchymal transition (EMT) and suppress the expression of the enhancer of zeste homolog 2 (EZH2) protein gene [26, 27]. By promoting cell apoptosis and inhibiting cell proliferation, EMO exerts an anti-renal fibrosis effect by modulating the TGF-β/Smad signaling pathway [28]. Additionally, Asiatic acid (AA), a pentacyclic triterpenoid compound, induces Smad7, further contributing to its anti-fibrotic effects [29–31]. Based on these observations, we hypothesized that the combination of EMO and AA may yield a more potent therapeutic effect on renal fibrosis by more effectively correcting the imbalance of TGF-β/Smad signaling. However, the oral administration of EMO and AA results in low bioavailability due to their susceptibility to glucuronidation metabolism and oxidative conversion, leading to limited accumulation in the kidney [32, 33]. Therefore, an efficient drug delivery system is required to enhance the renal targeting of EMO and AA.

Yeast cell wall particles (YCWPs) derived from *Saccharomyces cerevisiae* [34], commonly known as baker’s yeast, present natural carriers with porous and hollow structures ideal for cargo encapsulation [35, 36]. Comprising β-glucan, mannose, and chitin, YCWPs have been optimized as targeted carriers for various macrophages, including intestinal M cells macrophages, and other phagocytic cells [37, 38]. Due to their low toxicity, YCWPs are frequently utilized as biocompatible carriers to enhance the bioavailability of drugs and food products [39, 40].

In this study, we loaded EMO and AA onto YCWPs, enhancing their capacity to accumulate in the kidneys. However, certain channels on the YCWPs surface permit molecules with a molecular weight below 1000 Daltons to pass through, potentially resulting in drug leakage. To address this challenge, the scientific community has prioritized the transformation of small molecule drugs into supramolecular polymers [41, 42]. Molecular building blocks can self-assemble into supramolecular polymers through noncovalent synthesis, involving interactions like hydrogen bonding, π-π interaction, host–guest interaction, and metal–ligand interaction [43–45]. This approach increases the molecular weight, solubility, and drug retention [46]. In this study, we facilitated the self-assembly of EMO and AA into supramolecular nanoparticles, which were subsequently loaded onto YCWPs.

Increasing evidence points to a significant connection between renal fibrosis and disruptions in the composition of gut microbiota [47]. In the pursuit of regulating intestinal flora affected by renal fibrosis, various treatment options have emerged, including dietary adjustments, prebiotics, probiotics, and inhibitors of host and bacterial enzymes [48–51]. Probiotic pretreatment has demonstrated its ability to lower uremic toxin levels and decrease concentrations of urea and creatinine [52–54]. In animal models of kidney failure, the oral administration of a microbial cocktail effectively eliminates nitrogenous metabolic waste [55]. *Lactobacillus casei* Zhang (*L. casei* Zhang), isolated from traditional koumiss in Inner Mongolia, China, not only increases the levels of SCFAs and nicotinamide in both serum and kidneys but also ameliorates intestinal dysregulation, mitigates intestinal inflammation, and restores the integrity of intestinal epithelial tight junctions [56, 57]. However, a significant challenge in utilizing probiotics lies in ensuring their viability at the time of administration [58–60]. Biocompatible and biodegradable polymers, equipped with specific functional groups or sensitivity to pH/enzymes, have demonstrated potential as carriers for probiotics [61–63]. These polymers enhance probiotic viability, extend storage durations, shield against the harsh gastric environment, and enable precise delivery to target locations in the gastrointestinal tract.

The survival and colonization of *L. casei* Zhang in the complex and dynamic human gastrointestinal tract are inherently challenging [64]. To overcome these hurdles, protective measures are imperative to ensure safe passage through the acidic stomach environment and successful colonization in the colon [65]. Chitosan/alginate (CS/SA) microgels, characterized by their highly hydrated three-dimensional network, exceptional loading capabilities, and mechanical strength [66–68], represent one of the most widely employed microcapsule systems for encapsulating, safeguarding, and delivering probiotics [69, 70]. These microgels satisfy many of the essential requirements for effective probiotic delivery [71]. Therefore, we adopted a strategy where supramolecular yeast microparticles were co-loaded with probiotics onto CS/SA microgels for the purpose of efficient delivery.

**Scheme 1:**
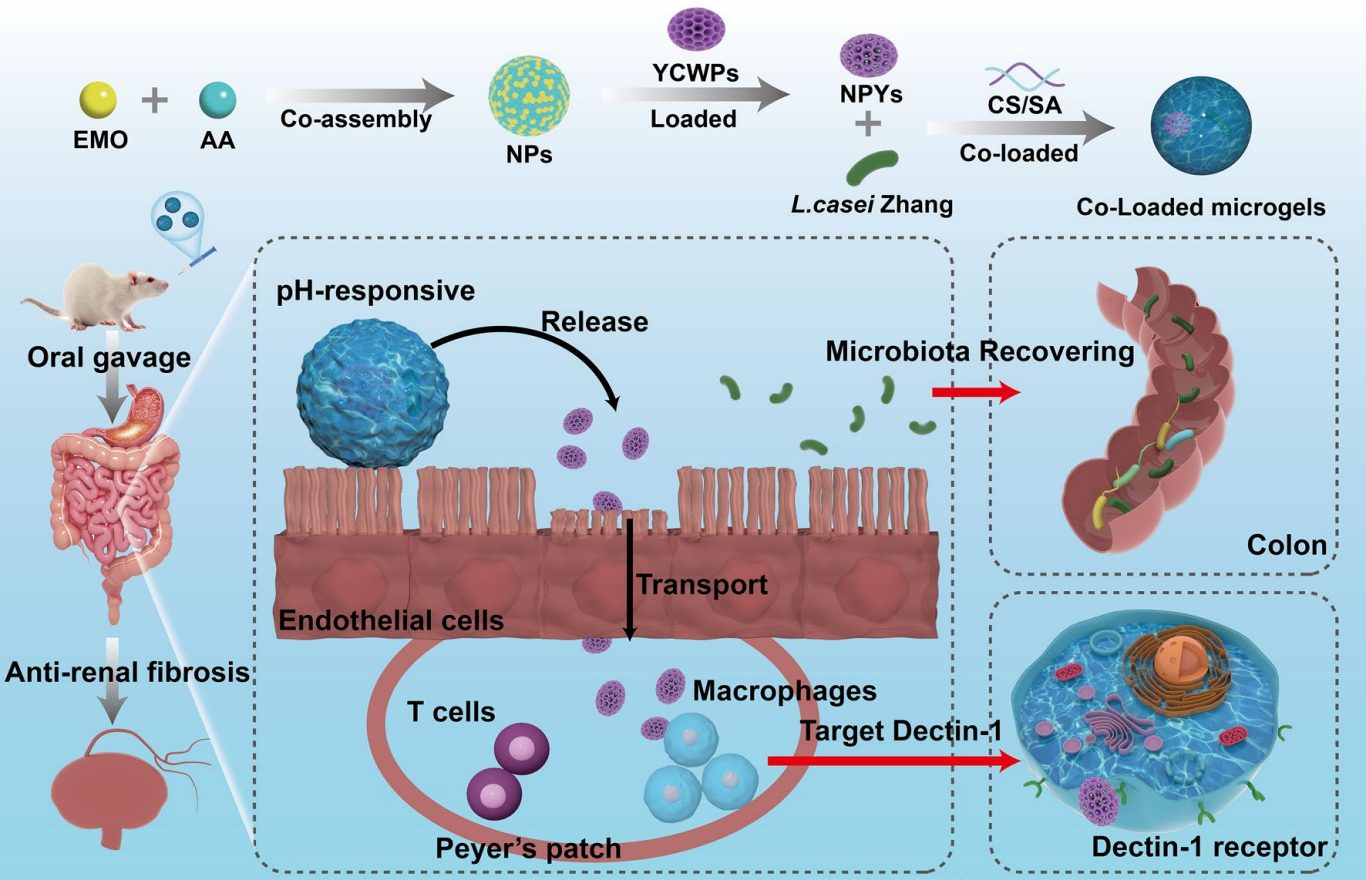
Schematic representation of microgels construction, Dectin-targeting, and microbiota recovering of Co-Loaded Microgels

This project aims to develop an integrated oral microgel system capable of delivering supramolecular yeast particles and probiotics to the intestinal tract (Scheme 1). This system can modulate intestinal flora and exert anti-renal fibrosis effects. EMO and AA were initially assembled and loaded into YCWPs to form NPYs, designed for enhancing the bioavailability via improving cellular uptake of both compounds. Subsequently, NPYs and *L. casei* Zhang were encapsulated within CS/SA microgels to protect *L. casei* Zhang from the harsh gastric conditions, therefore fostering their viability and ensuring colonization. The characteristics of NPYs and Co-Loaded Microgels with NPYs and *L. casei* Zhang were examined. Various in vitro experiments were conducted to assess the cellular uptake of NPYs, and microgels were comprehensively characterized. Eventually, the pharmacological effects of microgels were evaluated through in vivo experiments using the UUO rats. This multifaceted approach holds great potential for modulating intestinal flora and combating renal fibrosis, offering a promising avenue for CKD management.

## Materials and methods

### Materials

Emodin (EMO, > 98.0% purity) and chitosan (CS, MW 65 kDa) were purchased from Shanghai Yuanye Bio-Technology Co., Ltd. Polyvinylpyrrolidone K30 (PVP K30) and Asiatic acid (AA, > 98.0% purity) were purchased from Shanghai Aladdin Biochemical Technology Co., Ltd. *Lactobacillus casei* Zhang (*L. casei* Zhang) was kindly provided by Key Laboratory of Dairy Biotechnology and Engineering, Ministry of Education, Inner Mongolia Agricultural University. *Saccharomyces cerevisiae* was procured from Angel Yeast Co., Ltd (Yichang, China). Sodium alginate (SA) and formamide fluorescein-5-isothiocyanate (FITC) reagents were purchased from Sigma-Aldrich (St. Louis, MO, USA). Fetal bovine serum (FBS), Dulbecco’s modified eagle’s medium (DMEM) and Trypsin–EDTA (0.05%) were purchased from Gibco BRL (Gaithersburg, MD, USA). Primary and secondary antibodies against transforming growth factor-beta1 (TGF-β1), alpha-smooth muscle actin (α-SMA) and recombinant mothers against decapentaplegic homolog 2 (Smad2) were purchased from Abcam Biotech (Cambridge, MA, USA).

### Preparation and characterization of NPYs

The EMO/AA nanoparticles (NPs) were firstly prepared by a green and simple self-assembly approach, as reported previously with slight modifications [72]. Briefly, AA was dissolved in ethanol, and the solution was adjusted to pH 8.8 with 0.1 M sodium hydroxide. To this solution, the EMO (1.8 mM stock in ethanol) was added and stirred for 1 h at room temperature. The resulting mixture was gradually added to 10 mL of water containing 0.08% (w/v) PVP K30 as a stabilizer, which was stirred for 2 h at room temperature. Then, the ethanol was removed by dialyzing against deionized water for 12 h to obtain the NPs.

Subsequently, YCWPs were obtained from *Saccharomyces cerevisiae* using alkali and acid treatment methods, as described previously [73]. Afterward, the vacuum force-driven self-deposition with solvent hydration/lyophilization method was used to encapsulate the prepared NPs into the YCWPs [74]. Specifically, the YCWPs were dispersed in water with a concentration of 2 mg/mL upon stirring for 1 h at 37 °C. Then, an appropriate amount of NPs was added dropwise to the solution under continuous stirring for 2 h. After treatment under vacuum for 1 h at 0.08 MPa, lyophilization, the NPs@YCWPs (NPYs) were obtained. When noted, the YCWPs were fluorescently labelled with fluorescein isothiocyanate (FITC) as described previously [75] and EMO was partially replaced by doxorubicin (DOX) in the assembling of NPs [72, 76], because of the similar representative functional groups.

The morphology of different particles was visualized under transmission electron microscopy (TEM, 1200EX, Tokyo, Japan) and scanning electron microscopy (SEM, SU8010, Hitachi, Japan), respectively. The composite carriers, NYPs, were also observed by confocal laser scanning microscope (CLSM, Zeiss LSM510, Oberkochen, Germany) using the fluorescently-labelled samples.

Size and zeta potential of different particles were determined by a Zetasizer Nano ZS90 (Malvern Instruments, Malvern, UK). To determine encapsulation efficiency (EE) and drug loading (DL), the lyophilized NYPs, meticulously weighed, were diluted with 10 mL of methanol and centrifuged at 3000 rpm for 10 min, and the filtrate was injected into HPLC system. The concentration of EMO in the filtrate was measured by a previously validated high-performance liquid chromatography (HPLC) method at 250 nm [77]. To measure the concentration of AA, HPLC analysis was carried out on a Waters e2998 system consisting of a ZORBAX Extend-C18 (5 μm, 250 × 4.6 mm). 0.1% phosphoric and acetonitrile were the mobile phases and the gradient elution procedure was set to 0–25 min, 40–95% acetonitrile. The detection wavelength was set to 210 nm and the flow rate was 1 mL/min. EE and DL were calculated according to the following Eqs. (1) and (2):

1 $${\text{EE (\% ) = }}\left( {\text{amount of drug in particles/total amount of drug added}} \right) \times {100}$$

2 $${\text{DL (\% ) = }}\left( {\text{amount of drug in particles/weight of particles}} \right) \times \,{100}$$

### Uptake of NPYs by macrophages

RAW 264.7 cells, derived from the Cell Bank of Chinese Academy of Science (Shanghai, China), were cultured in DMEM supplemented with 10% (v/v) fetal bovine serum (FBS), 1% (v/v) penicillin and 1% (v/v) streptomycin in an incubator at 37 ℃ and 5% carbon dioxide. The cells were seeded in confocal dishes at a density of 2 × 10^5^ cells per well to adhere for 24 h. Then, the fluorescence-labeled NPs and NPYs were introduced in each well at the DOX concentration of 15 μg/mL, respectively. After 4 h, cells were washed and fixed with 4% paraformaldehyde, and followed by 4′,6-Diamidino-2-phenylindole (DAPI) staining before being visualized under CLSM [36]. To investigate the involvement of Dectin-1 in endocytic processes, cells were pretreated with laminarin (1 mg/mL), a Dectin-1 competitor, for 30 min prior to the addition of fluorescence-labeled NPs and NPYs.

For flow cytometry analysis, the RAW 264.7 cells were seeded in 6-well plates at a density of 2 × 10^5^ cells per well to adhere for 24 h, and incubated with fluorescence-labeled NPYs for 4 h at the DOX concentration of 30 μg/mL. Subsequently, the cells were rinsed with cold phosphate buffered saline (PBS), resuspended, and analyzed using a flow cytometer (CytoFlex, Beckman, USA) with FlowJo 10.8.1 software (v10 version, BD Biosciences, Ashland, OR, USA).

### Loading NPYs and L. casei Zhang into microgels

To fabricate the microgels, a simple device was made by ourselves, which was composed of a high-voltage pulse device, an adjustable voltage knob, a metal copper coil and a metal needle with a size of 340 μm. To initiate the process, the prepared NPYs and 10 mg of dry powder of *L. casei* Zhang (10^8^ CFU/mg) were firstly suspended in deionized water and sterilized saline, respectively. Afterward, they were added to a SA solution (2%, w/v). The mixture was then left to stand for 5 min to allow any air bubbles to leave the system. Then, the mixture was transferred into the syringe in the self-designed device and injected into a solution containing 1% CaCl_2_ and 0.05% CS at the rated voltage. Finally, let the microgels remain for 10 min, rinse thrice with deionized water, the microgels were collected, lyophilized, and stored at 4 °C.

### Characterization of the microgels

An inverted fluorescence microscope (TI-s, Nikon, Japan) was used to observe the obtained microgels and size distribution of the microgels was analyzed by using Image J 2022 software. Zeta potential of the microgels was determined by a Nano-ZS90. The morphology of the microgels was analyzed using SEM. For fourier transform infrared spectroscopy (FT-IR) analysis, SA, CS, Blank Microgels and Co-Loaded Microgels were pressed with KBr and scanned from 4,000 to 400 cm^−1^ to confirm the interaction between SA and CS.

EE and DL of EMO and AA in microgels were determined by HPLC. Lyophilized microgels were diluted with methanol, sonicated using a sonicator (SCIENTZ-IID, Xin Zhi, China), centrifuged at 3000 rpm for 10 min, and the filtrate was injected into a HPLC system to quantify the amount of EMO and AA as described above. The EE and DL of *L. casei* Zhang in microgels were firstly determined using methylthiazolyldiphenyl-tetrazolium bromide (MTT) assay [78]. Briefly, the lyophilized microgels were firstly dispersed in water, followed by addition of methanol. Then the mixture solution was vortexed and sonicated for 10 min. 100 μL solution was aspirated and mixed with 20 μL of MTT solution and then incubated at 37 ℃ for 2 h, followed by addition of 100 μL of dimethyl sulfoxide (DMSO) solution. After thorough mixing, the absorbance value at 570 nm was measured. Moreover, flow cytometry (FCM) was also used to determine the EE and DL of *L. casei* Zhang in microgels. Briefly, the encapsulated *L. casei* Zhang was firstly released by dissolving the beads in 10% sodium citrate [79]. Then, the collected cells were stained using the live/dead backlight bacteria viability kit and then analyzed with FCM. The EE and DL were calculated using the following Eqs. (3) and (4):

3 $${\text{EE (\% ) = (number of probiotics in microgels/number of probiotics added)}} \times {100}$$

4 $${\text{DL (\% ) = (number of probiotics in microgels/weight of microgels)}}\, \times \,{100}$$

### In vitro release and swelling assessment

To begin, 50 mg of lyophilized microgels was initially mixed with 50 mL of simulated gastric fluid (SGF, pH 1.2, containing 16.4 mL dilute hydrochloric acid and 10 g pepsin per 1 L water), which were placed in an oscillator (ZD-85, Lichen, China) with continued shaking at 37 °C [80]. After 2 h, microgels were collected and incubated in 50 mL simulated intestinal fluid (SIF, pH 6.8, containing 6.8 g potassium dihydrogen phosphate and 10 g pepsin per 1 L of water and adjusting with 0.1 M sodium hydroxide to pH 6.8) for 6 h, and 50 mL simulated intestinal fluid (SIF, pH 7.4, purchasing from Wokai Biotechnology Co., Ltd) for incubation for 16 h successively. Then, 1 mL of sample was withdrawn at predetermined time intervals and replaced with equal volume of fresh medium.The contents of EMO, AA, and the *L. casei* Zhang in the samples were determined using HPLC method and MTT assay respectively, as mentioned above.

The swelling characteristic of the unfilled microgels was evaluated in various pH of buffer solution (pH 1.2, 2.5, 5.0, 6.8, 7.0, and 7.4) [81]. In brief, a predetermined quantity of lyophilized microgels was meticulously weighed (W_0_) and immersed in 2 mL of various media at 37 ℃, respectively. At predetermined time intervals, the swollen microgels were extracted from the media, gently wiped to eliminate excess surface water, and reweighed (W_t_). Moreover, morphological changes of the microgels in different media were observed by SEM. The swelling ratio (% SR) was calculated using equation as follows:

5 $${\text{\% SR = }}\left[ {{\text{(W}}_{{\text{t}}} {\text{ - W}}_{{0}} {\text{)/W}}_{{0}} } \right] \times \,{100}$$

where W_0_ is the initial weight of microgels and W_t_ is the weight of microgels in swollen state after incubation in PBS at predetermined time interval.

### Distribution of microgels

All animal experiments were conducted in accordance with the approved guidelines for the use and care of animals established by the Animal Ethical and Welfare Committee of Zhejiang Chinese Medical University (License No. SCXK 2019–0024, Hangzhou, China). To explore the distribution of different formulations in mice’ gastrointestinal tract (GIT), a total of 45 BALB/c mice were randomly divided into three groups (n = 3) and administered intragastrically with DOX-labelled NPs, DOX-labelled NPYs, and DOX-labelled microgels at DOX dose of 2 mg/kg, respectively [82, 83]. The stomach and intestines were collected at predetermined time points for bioluminescence imaging using the VISQUE In Vivo Elite (VIEWORKS, Korea) imaging system.

Additionally, the distribution of the microgels in the Peyer’s patches and organs including heart, lung, liver, spleen, normal kidney, and UUO kidney were explored. Briefly, 12 BALB/c mice were anesthetized intraperitoneally with 1% pentobarbital at a body weight of 40 mg/kg and underwent UUO operations as previously described [84]. After recovering from the surgery, these UUO mice were divided randomly and administered intragastrically with FITC-YCWPs or FITC-YCWPs-Microgels at FITC dose of 2 mg/kg. At specific time points, the Peyer’s patches of UUO mice administered with FITC-YCWPs-Microgels were extracted, fixed with 4% paraformaldehyde. Subsequently, the samples were sectioned at 20 μm using a freezing microtome. Cryosections were air-dried at room temperature for 24 h before immunostaining, stained with DAPI, and photographed by CLSM. Moreover, the heart, lung, liver, spleen, normal kidney, and UUO kidney of mice administered with FITC-YCWPs and FITC-YCWPs-Microgels were collected at different time points and photographed by VISQUE In Vivo Elite imaging system.

### Therapeutic effect against UUO model

SD rats with an initial weight of 200 (± 20) g were used in this experiment. After being anesthetized intraperitoneally with 1% pentobarbital at a body weight of 60 mg/kg, the UUO and Sham operations were performed according to our previously established protocol [84]. Model and sham groups were treated with saline, Others were divided into benazepril (BNPL) group (administered intragastrically with BNPL at a dose of 10 mg/kg), EMO&AA group (administered intragastrically with the mixture of EMO and AA at EMO dose of 10 mg/kg and AA dose of 20 mg/kg) [85, 86], Drug-Loaded Microgels group (administered intragastrically with NPYs-loaded microgels at EMO dose of 10 mg/kg and AA dose of 20 mg/kg) and Co-Loaded Microgels group (administered intragastrically with NPYs and probiotics Co-Loaded Microgels at EMO dose of 10 mg/kg, AA dose of 20 mg/kg and *L. casei* Zhang dose of 10^9^ CFU/per mouse), followed by administration with corresponding formulations for 21 consecutive days. The body weight of each rat was recorded every day. On day 22, fresh feces and blood samples from each rat were collected and stored at −80 ℃. Afterward, the rats were sacrificed via CO_2_ asphyxiation, and the obstructed kidney and colon were harvested for histological, immunohistochemical (IHC), and western blot analysis, respectively. The hematological and biochemical parameters were measured by using enzyme-linked immunosorbent assay (ELISA) kits (Jiangsu Meimian Industrial Co., Ltd, Yanchen, China). 16S rDNA sequencing analysis of gut microbiota was performed by LC-Bio Technology Co., Ltd (Hangzhou, China).

### Statistical analysis

All data were expressed as means ± standard deviation. GraphPad Prism 9.5 software (GraphPad, CA, United States) and SPSS software package (version 24.0, IBM Inc., USA) were used for statistical analysis. Student’s T-test was used for comparison between two groups, and one-way ANOVA was used where more than two groups were compared. Differences were considered to be statistically significant when ^*^P < 0.05; ^**^P < 0.01; ^***^P < 0.001; ^****^P < 0.0001.

## Results and discussion

### Formation and characterization of NPYs

**Fig. 1:**
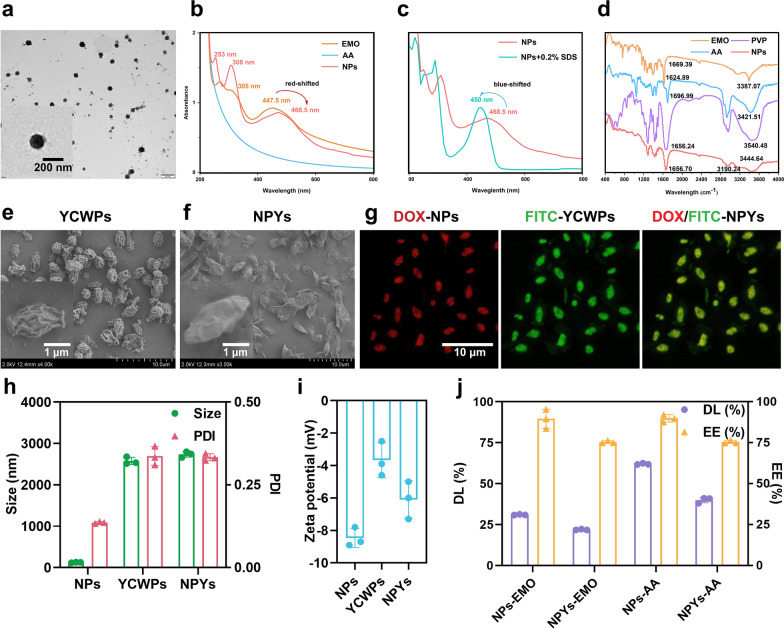
Characterization of different particles. **a** TEM image of the NPs. **b**,** c** UV absorption spectra of EMO, AA, NPs, and NPs + 0.2% SDS. **d** FT-IR spectra of EMO, AA, PVP, and NPs. **e**,** f** SEM images of blank YCWPs and NPs loaded YCWPs (NPYs). **g** Representative confocal images of DOX-labelled NPs (red) @YCWPs with FITC labeling the yeast cell shell (green). **h**,** i** Particle size, polydispersity index (PDI), zeta potential of the NPs and the NPYs (mean ± SD, n = 3). (**j**) EE and DL of the NPs and the NPYs (mean ± SD, n = 3)

The NPs were formed by co-assembling deprotonated AA and EMO without the use of additional carrier materials. The NPs exhibited a spherical morphology (Fig. 1a) and a uniform size distribution, with an average particle size of 124.0 ± 4.30 nm and a PDI of 0.136 ± 0.003 (Fig. 1h). The NPs possessed a negative zeta potential (approximately–8.5 mV (Fig. 1i), which could be attributed to the deprotonation of AA using sodium hydroxide before co-assembly with EMO [72]. This deprotonation process is crucial for the nanoassembly of carrier-free nanodrugs through supramolecular interactions, including electrostatic, hydrophobic, and π-π stacking interactions. Upon deprotonation with sodium hydroxide, the carboxyl group of AA becomes hydrophilic, thus, balancing the hydrophilicity and hydrophobicity within the system. The EE and DL values of EMO and AA in the NPs were 89.5 ± 6.51%, 90.0 ± 2.54% and 30.9 ± 0.84%, 61.9 ± 0.62%, respectively (Fig. 1j).

Previous studies have demonstrated that EMO molecules adopt a rod-like structure in aqueous solutions (Fig. S1a). As a bile salt and bile salt analog, AA, a pentacyclic triterpenoid, possesses amphipathic properties and behaves like a surfactant-like cosolvent, leading to self-assembly into nanoarchitectures through π-π stacking and hydrophobic interactions [87]. Under TEM, the self-assembled AA exhibits a wire-like morphology (Fig. S1b), consistent with previous descriptions. When EMO is introduced for co-assembly with AA, uniform-sized spherical NPs are obtained (Fig. S1c). Interestingly, the morphology and size of the NPs depend on the molar ratio of EMO to AA molecules, which regulates the hydrophilic/hydrophobic balance of the NPs system, thereby modulating the shape and size distribution of the resulting assemblies [88]. Various assemblies, including rod-like, sphere-like, and wire-like structures, can be obtained (Fig. S2), depending on the EMO/AA ratios ranging from 2:1, 1:1, 1:2, to 1:3. The uniformly sized spherical NPs with well-monodispersed size distribution are obtained at a molar ratio of 1:1 (EMO to AA), which falls within the range of synergistic therapeutic ratios of EMO and AA (Fig. S3). Additionally, PVP is employed to enhance the stability and dispersibility of the NPs system, facilitated by strong hydrogen bonding interactions between the phenolic hydroxyl groups and PVP [89].

The co-assembly mechanism of the NPs was confirmed through ultraviolet (UV) spectra and FT-IR analysis. Supramolecular assemblies typically involve non-covalent interactions, such as hydrogen bonding, π-π stacking, and hydrophobic interactions [90]. The UV spectra showed broader and red-shifted absorbance peaks upon the co-assembly of EMO with AA (Fig. 1b). This observation suggests a molecular conformational change caused by the π → π* transitions of EMO with AA [91, 92]. Furthermore, when the NPs were incubated with sodium dodecyl sulfate (SDS) at a concentration of 0.2% w/v, a blue shift in the UV spectra of the NPs was observed, indicating disassembly of the NPs, likely due to the induction of additional hydrophilic interactions by SDS (Fig. 1c) [93]. FT-IR data also supported the co-assembly of EMO with AA (Fig. 1d) by the shift of the characteristic absorption bands for EMO and AA, such as C = O and –OH, indicating the presence of hydrogen bonding between the unsaturated cycloalkanes in AA and the phenolic hydroxyl groups in EMO. Based on these findings, it can be reasonably concluded that the successful co-assembly of EMO with AA molecules was primarily attributed to the hydrogen bonding, π-π stacking, and hydrophobic interactions.

The next step involved the characterization of NPYs. SEM imaging revealed that YCWPs exhibited a regular ellipsoidal or ovoid shape with a diameter of 2 ~ 4 µm (Fig. 1e). The surface of the YCWPs displayed a porous structure, which was well-suited for encapsulating various NPs (Fig. 1e). In contrast to the YCWPs, the surface of the NPYs appeared smooth rather than rough (Fig. 1f), indicating that the NPs were retained within the deep channels of the YCWPs. This observation was further confirmed by CLSM imaging (Fig. 1g). The EE and DL values of EMO and AA in the NPYs were 75.4 ± 0.80%, 75.4 ± 0.90% and 21.9 ± 0.30%, 40.0 ± 1.70%, respectively (Fig. 1j).

### Uptake of NPYs by macrophages

**Fig. 2:**
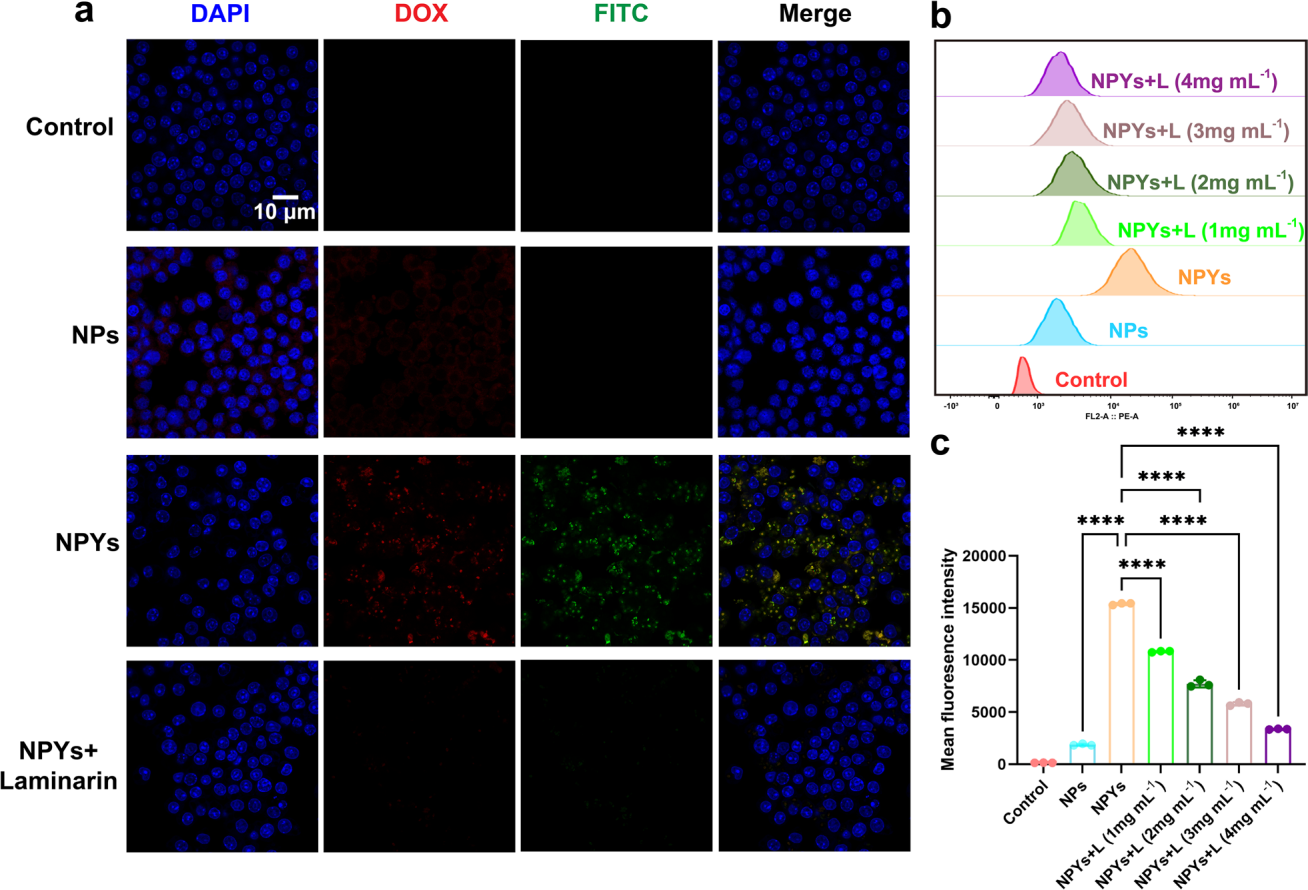
Cellular uptake of particles by RAW 264.7 macrophages. **a** Representative confocal images of RAW 264.7 cells incubated with NPs, NYPs, NPYs + laminarin for 4 h, respectively. **b**,** c** Flow cytometry of RAW 264.7 cells treated with NPYs in the presence of laminarin at different concentrations (1, 2, 3, 4 mg/mL) (mean ± SD, n = 3) (****P < 0.0001)

We then investigated the interactions between NPYs and macrophages. To directly compare the cellular uptake efficiency of NPs and NPYs, NPs were labeled with DOX, while YCWPs were labeled with FITC. CLSM analysis revealed that the fluorescence intensity of RAW 264.7 cells incubated with fluorescence-labeled NPYs was significantly stronger than that of NPs (Fig. 2a). Furthermore, when the cells were pre-incubated with laminarin, a β-glucan antagonist, the fluorescence intensity of RAW 264.7 cells treated with fluorescence-labeled NPYs markedly decreased [94]. Having confirmed the internalization of NPYs by RAW 264.7 cells, we further evaluated the cellular uptake using FCM to ascertain whether β-glucan, the main component of YCWPs, facilitates the uptake of NPYs by specifically binding to Dectin-1 receptors expressed on macrophages [40]. Therefore, RAW 264.7 cells were pre-incubated with various concentrations of laminarin before the addition of NPYs for FCM analysis. As demonstrated in Fig. 2b, c, the recognition and phagocytosis of NPYs by macrophages were largely associated with Dectin-1 receptors.

### Formation and characterization of microgels

**Fig. 3:**
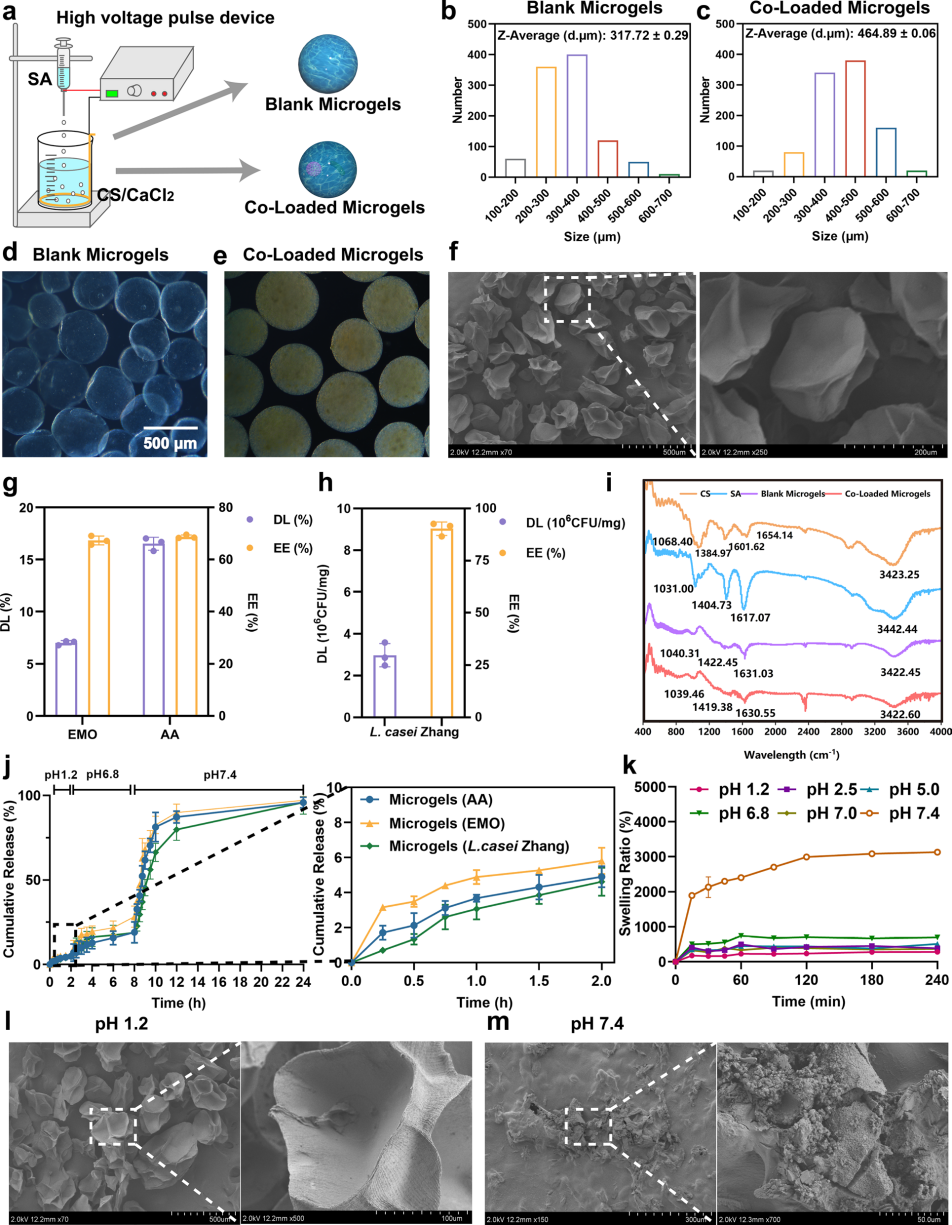
Characterization and in vitro evaluation of microgels. **a** Schematic diagram of fabrication of microgels. **b**,** c** Particle size distribution of Blank Microgels and Co-Loaded Microgels. **d**,** e** Light microscope diagram of Blank Microgels and Co-Loaded Microgels. **f** SEM images of Co-Loaded Microgels and higher magnification images of the white-outlined area. **g**,** h** Drug loading (DL) and encapsulation efficiency (EE) of the microgels (mean ± SD, n = 3). **i** FT-IR spectra of CS, SA, Blank Microgels and Co-Loaded Microgels. **j** In vitro release of EMO, AA, and *L. casei* Zhang from microgels in different media and higher magnification images of the black-outlined area. **k** Swelling behavior of microgels in different media. **l**,** m** SEM images of microgels in SGF (pH 1.2), and SIF (pH 7.4) and higher magnification images of the white-outlined area, respectively

A self-designed device consisting of a high voltage pulse device, adjustable voltage knob, metal copper coil, and metal needle was utilized to generate CS/SA microgels with and without encapsulation of NPYs and *L. casei* Zhang (Fig. 3a). It was observed that the voltage generated during the preparation process had no effect on the viability of the probiotics (Fig. S4a). Also, the loading of NPs into the YCWPs prevents their direct contact with probiotics, which, in turn, maintains the viability of *L. casei* Zhang. (Fig. S4b). Moreover, CLSM images and FCM analysis indicated that probiotic cells were evenly distributed inside the microgels (Fig. S5a-c), and the encapsulation of NPYs within the microgels barely influences the viability of *L. casei* Zhang.

The prepared Blank Microgels and Co-Loaded Microgels appeared as spherical shapes under inverted fluorescence microscope (Fig. 3d, e). The average particle diameters of the Blank Microgels and Co-Loaded Microgels were determined to be 317.72 ± 0.29 µm and 464.89 ± 0.06 µm, respectively (Fig. 3b, c). The increased size of the microgels after incorporating NPYs and *L. casei* Zhang indicates that these additives influenced microgels formation during the injection process. The SEM images revealed that the lyophilized microgels had a regular spherical shape, and their surfaces appeared shrunken and wrinkled (Fig. 3f). Moreover, the diameter of microparticle was reduced from 464.89 µm to approximately 200 µm after drying process. It happens due to the high water content in microgels, ranging from 70 to 90% [95]. The EE and DL values of EMO and AA in the microgels were 67.31 ± 2.51%, 68.75 ± 1.01%, and 7.03 ± 2.92%, 16.52 ± 3.74%, respectively (Fig. 3e). The DL of *L. casei* Zhang was 2.98 ± 0.56 × 10^6^ CFU/mg (Fig. 3g, h).

To verify the microgels gelling processes and structure formation, the FT-IR spectra of CS, SA, Blank Microgels and Co-Loaded Microgels were analyzed (Fig. 3i). In the CS molecule, the stretching vibration of –OH and –NH was observed at 3423.25 cm^–1^, while the stretching vibration of –C = O and the bending vibration of –NH were observed at 1654.14 cm^–1^ and 1601.62 cm^–1^, respectively. Additionally, the out-of-plane bending vibration of –CH_2_ was located at 1384.97 cm^–1^, and the stretching vibration of –C–O was located at 1068.40 cm^–1^. The vibrational frequencies of SA (–OH stretching, –C = O symmetry, asymmetric stretching and –CN stretching) were observed at 3442.44 cm^–1^, 1617.07 cm^–1^, 1404.73 cm^–1^ and 1031.00 cm^–1^. In Blank Microgels, characteristic peaks of CS and SA were detected at 1631.03 cm^–1^ and 3422.45 cm^–1^. The absence of peak at 1601.62 cm^–1^ should be attributed to protonation of –NH_2_ into –NH^3+^, while the shift of SA peaks from 1404.73 cm^–1^ to 1422.45 cm^–1^ suggests an electrostatic interaction between –COO^–^ in SA and –NH^3+^ in CS [96]. Furthermore, the overlapping absorption peaks formed by the carbonyl group shifted to lower wavenumbers, providing further evidence of the strong electrostatic interaction between the amino group in the CS molecular chain and the carboxylate functional group in the SA molecular chain [97]. Additionally, the encapsulation of NPYs and probiotics barely affects the structural properties of the microgels.

### Release behavior and swelling of microgels

The release of EMO, AA, and *L. casei* Zhang from the Co-Loaded Microgels was evaluated in different media (Fig. 3j). In SGF (pH 1.2), the release of EMO, AA, and *L. casei* Zhang from microgels was minimal because of the restricted swelling capacity of microgels. However, upon transfer of the microgels to SIF, the release of EMO, AA, and *L. casei* Zhang significantly increased, especially their release in SIF (pH 7.4) increased even more with the cumulative release of 95% within 24 h. It is worth noting that the pH in the small intestine gradually increases from pH 6 in the duodenum to about pH 7.4 in the terminal ileum where Peyer’s patches are located. The release behavior of drugs and *L. casei Zhang* from the microgels at pH 7.4 indicated that the microgels are efficient in protecting probiotics from harsh conditions in the gastrointestinal tract while facilitating the complete release of NPYs in the terminal ileum, which enhances the delivery of NPYs into Peyer’s patches. Additionally, The release behavior aligns with the swelling properties of the microgels. The microgels exhibited a swelling rate of 200% at pH 1.2, which remarkably increased to 3000% at pH 7.4 (Fig. 3k). Under an acidic environment, microgels retained their initial morphology as a result of the protonation of the CS’s and SA’s –COO^–^ groups to –COOH, a large number of hydrogen bonding and intermolecular electrostatic interactions make the gel network closer [98]. The swelling behavior was greatly enhanced in high pH media, as –COOH dissociates to –COO^–^ due to the negative charge, resulting in repulsive force lose [99]. Consequently, electrostatic interaction between SA and CS was diminished, facilitating the process of water absorption and swelling of alginate. This phenomenon was further confirmed by SEM imaging, which revealed the pH-induced degradability of the microgels at pH 7.4 (Fig. 3l, m).

### Biodistribution of microgels

**Fig. 4:**
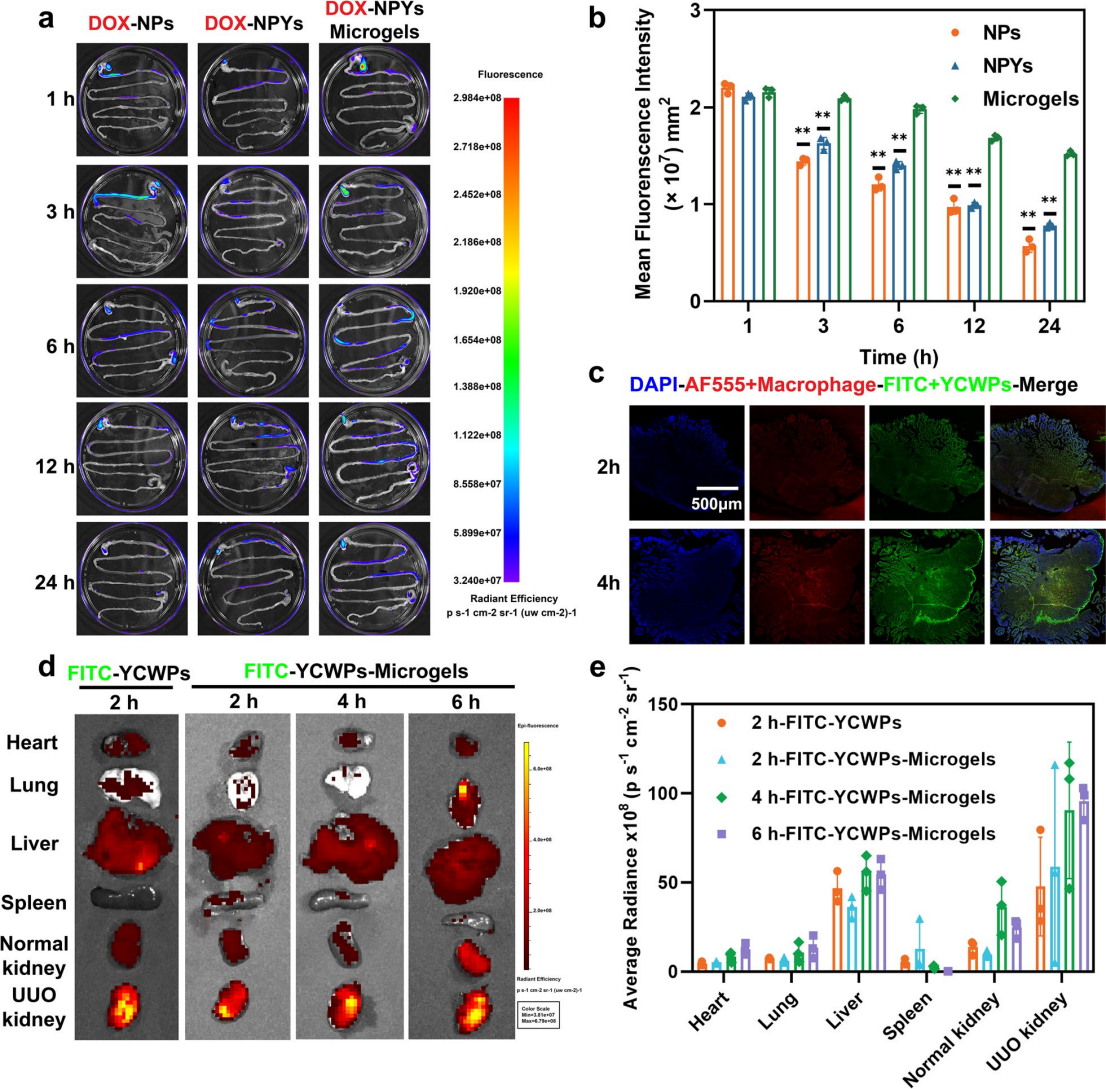
Biodistribution of microgels. **a**,** b** The isolated GIT fluorescence images and fluorescence signal histogram of different formulations at different time points. **c** Confocal images of Peyer’s patches at different times after UUO mice being gavaged with YCWPs-Microgels. **d**,** e** Time-dependent fluorescence signals of FITC that accumulated in isolated major organs after mice being gavaged with YCWPs or YCWPs-Microgels and their corresponding average radiances (mean ± SD, n = 3) (**P < 0.01)

The gastrointestinal retention of the microgels was further assessed. The biodistribution of NPs, NPYs, and microgels in GIT over time after oral administration is depicted in Fig. 4a, b. Within the first hour following oral administration, there was substantial retention of NPs, NPYs, and microgels in the stomach. Over time, an increased retention was observed in the intestine. Notably, microgels exhibited consistent accumulation in the intestine, which was clearly visible in all the images at 24 h. In contrast, the retention of NPs coacervate in the GIT was limited after 12 h. The bioadhesive nature and liquid state of CS/SA microgels enable adaptation to the complex motility, fluid content, and significant pH variations within the GIT [100]. This allows for sufficient intestinal retention, facilitating sustained drug release and colonization of probiotics.

The in vivo imaging system (IVIS) images of tissues showed that the FITC fluorescence signal from the UUO kidney was significantly stronger than that from the normal kidney on the opposite side of ligation (Fig. 4d), and the fluorescence intensity increased overtime (Fig. 4e). This suggests increased accumulation of FITC-YCWPs in UUO kidney. To explore the mechanism of their renal accumulation, the distribution of the FITC-YCWPs-Microgels in the Peyer’s patches of the mice at 2 h and 4 h post administration was examined. Obviously, the fluorescence intensity of the FITC-YCWPs (green) was very strong (Fig. 4c), not only spreading to the interstitial space but also permeating into the tubular structures. It provides evidence of the uptake of the NPYs by Peyer’s patches, potentially through M cells situated in the follicle-associated epithelium (FAE) [36, 94]. Moreover, a substantial amount of green fluorescence was detected in the vicinity of macrophages, suggesting the YCWPs could be recognized by Dectin-1 receptors [93], on macrophages, which was consistent with our in vitro data.

### Therapeutic efficacy of microgels against renal fibrosis

**Fig. 5:**
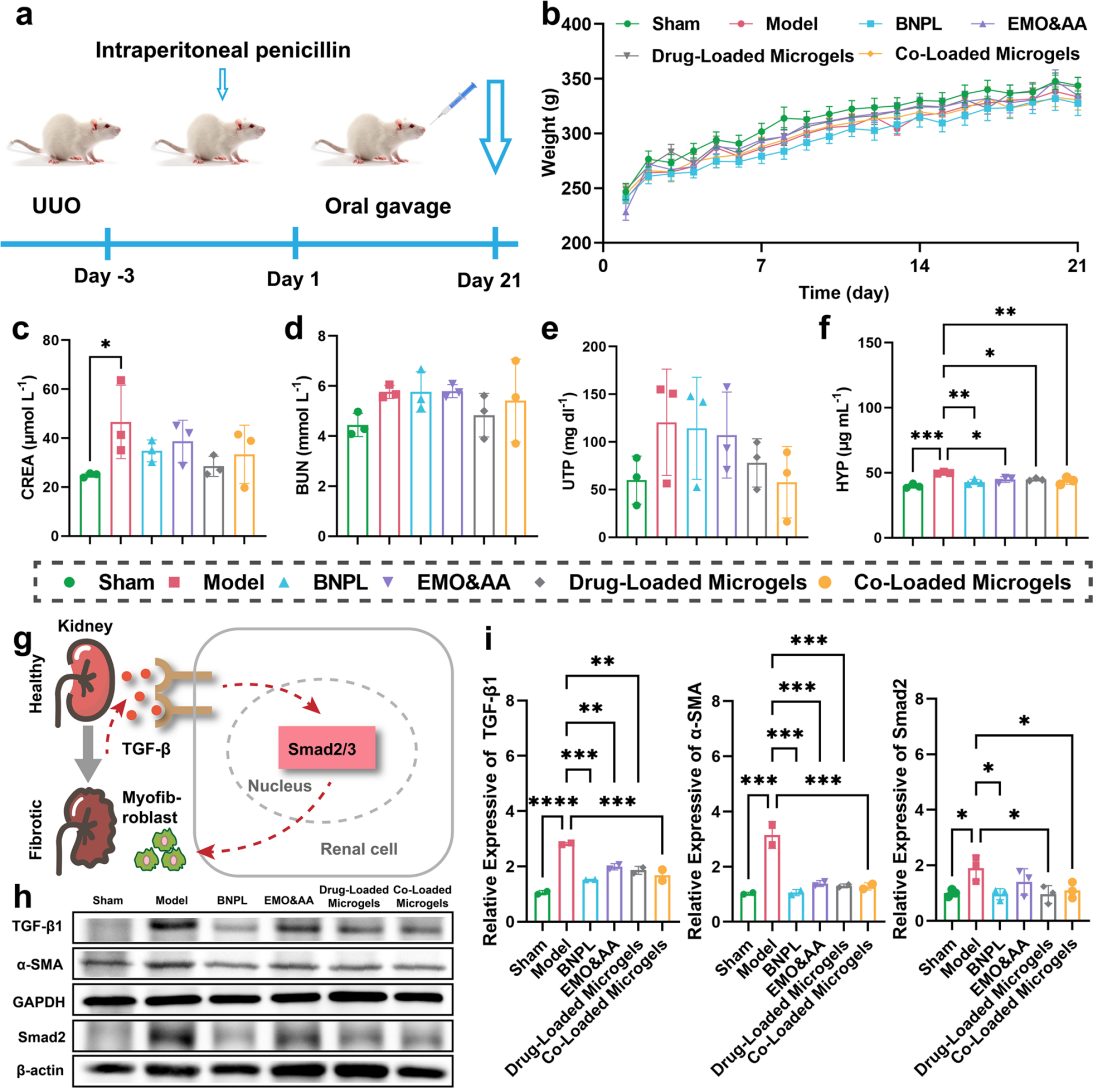
Combination therapy attenuated UUO-induced renal fibrosis in rats. **a** Schematic diagram of the establishment of UUO model and experimental protocol. **b** Body weight variation of UUO rats during the treatment period. **c**-**f** Creatinine (CREA), blood urea nitrogen (BUN), total urinary protein (UTP) and hydroxyproline (HYP) concentrations in each group. **g** Schematic diagram of the TGF-β/Smad signaling pathway. **h**,** i** Representative bands and fold changes of TGF-β, α-SMA, and Smad2 in each group determined by western blotting (mean ± SD, n = 3) (*P < 0.05, **P < 0.01, ***P < 0.001, ****P < 0.0001)

The experimental model is illustrated in Fig. 5a. Rats in sham group and each administration group exhibited normally, responsive behavior, normal food intake, and normal weight growth (Fig. 5b). No significant changes were observed in blood urea nitrogen (BUN) and total urinary protein (UTP) levels. However, the creatinine (CREA) levels in model group exhibited a significant increase in comparison to sham group (Fig. 5c) (*P* < 0.05), indicating reduced glomerular filtration in model group and successful establishment of the model [101]. The levels of BUN and UTP were within the normal range (BUN: 2.5–8.0 mmol/L; UTP: < 150 mg/d) [102], suggesting gradual recovery of glomerular filtration [103]; thus, no significant differences were observed among different administration groups. (Fig. 5d, e). In comparison to model group, the hydroxyproline (HYP) levels in all administration groups, particularly Co-Loaded Microgels group, were significantly decreased (Fig. 5f) (*P* < 0.001), indicating that the Co-Loaded Microgels effectively reduced collagen accumulation, which contributes to the attenuation of renal fibrosis in UUO rats [104].

To investigate the effect of microgels on TGF-β/Smad signaling pathway in UUO kidneys (Fig. 5g), western blot analysis was conducted to assess the expression of related proteins and the mesenchymal markers, such as α-SMA, TGF-β1, and Smad2 [105]. As shown in Fig. 5h, i, the protein expression of α-SMA, TGF-β1 and Smad2 were significantly elevated in model group compared to sham group. However, the upregulation of α-SMA, TGF-β1, and Smad2 induced by UUO surgery was effectively attenuated by all formulations. Among them, Drug-Loaded Microgels and Co-Loaded Microgels showed superior effects particularly. This suggests that EMO and AA attenuate renal fibrosis via modulation of TGF-β/Smad signaling pathway.

**Fig. 6:**
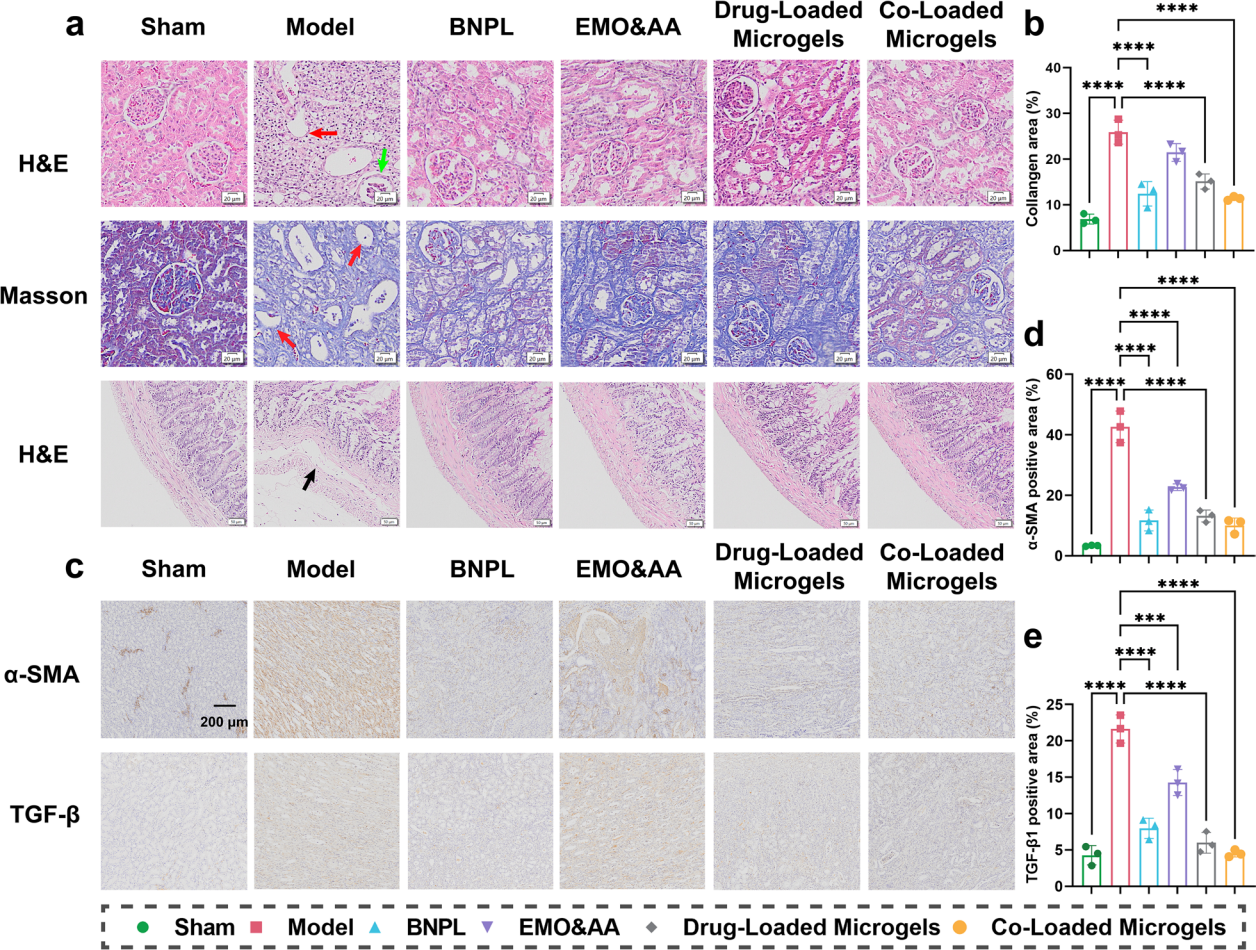
Pathology imaging of rats’ UUO kidney and intestine after UUO rats being administered with different formulations. **a** H&E, Masson staining of UUO kidney tissue and intestinal tissue of rats. **b** Collagen area in UUO rats after treatment with different preparations (mean ± SD, n = 3) (****P < 0.0001). **c** IHC staining of UUO kidney tissue of rats. **d**,** e** Semi-quantitative analysis of a-SMA, Collagen I, TGF-β in UUO rats after treatment with different preparations (mean ± SD, n = 3) (***P < 0.001, ****P < 0.0001)

To evaluate the effects of each formulation on renal fibrosis, histological staining with Hematoxylin–eosin (H&E) and Masson’s Trichrome was performed. Compared to sham group, H&E staining of UUO kidney tissue showed inflammatory cell infiltration, thickening of the basement membrane (indicated by green arrow), enlargement of the renal tubular lumen (indicated by red arrow), and interstitial fibrosis. As expected, drug interventions notably alleviated interstitial fibrosis (Fig. 6a). Notably, Co-Loaded Microgels group exhibited greater effectiveness than other formulations in attenuating inflammatory cell infiltration and reducing the deposition of extracellular matrix, and Masson’s staining (Fig. 6a, b) showed a similar trend. H&E staining of the colon in sham group showed no significant histopathological changes (Fig. 6a). However, in model group, local necrosis and perforation were observed in the submucosa, the muscular layer was thickened and delaminated (indicated by the black arrow), and the crypt structure was incomplete. In each drug administration group, the damage was reduced, and the colonic mucosa and crypt structure were relatively well-preserved.

IHC staining was conducted to assess the levels of α-SMA and TGF-β in UUO kidney tissues (Fig. 6c). The results demonstrated a significant increase in α-SMA and TGF-β expression in model group compared to sham group (Fig. 6d, e). In contrast, all treatment groups exhibited lower levels of α-SMA and TGF-β. Notably, Drug-Loaded Microgels and Co-Loaded Microgels group resulted in a more pronounced reduction of α-SMA and TGF-β than EMO&AA group, indicating the effective in against renal fibrosis.

**Fig. 7:**
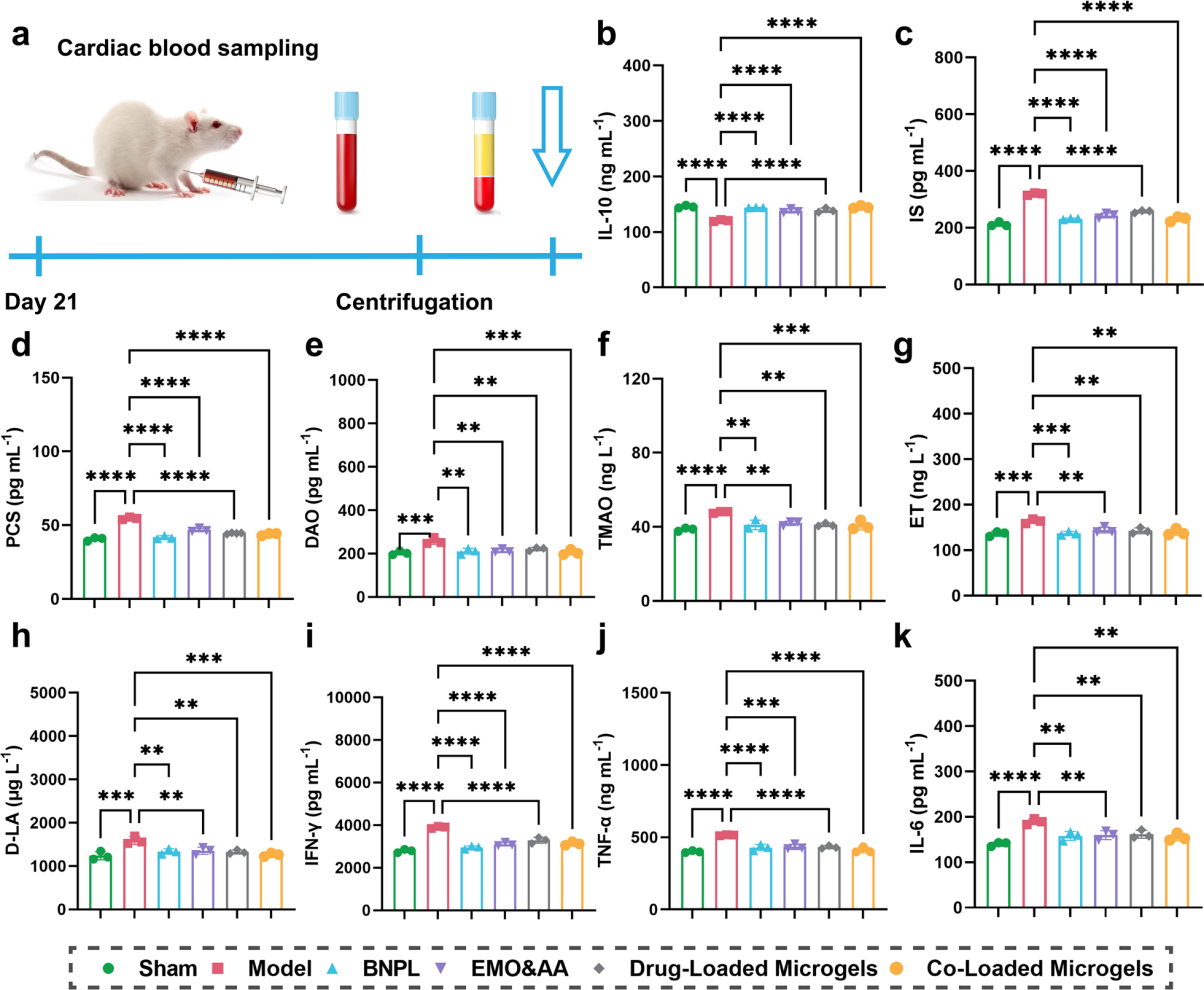
Intestinal injured biomarkers and uremic toxins in rats’ serum after UUO rats being administered with different formulations. **a** Schematic illustration of the cardiac blood sampling. **b** Serum levels of IL-10. **c**-**h** Serum levels of IS, PCS, DAO, TMAO, ET, and D-LA. **i**-**k** Serum levels of IFN-γ, TNF-α and IL-6 (n = 3, mean ± SD). (*P < 0.05, **P < 0.01, ***P < 0.001, ****P < 0.0001)

Additionally, changes in the levels of interleukin-10 (IL-10), indoxyl sulfate (IS), p-Cresol sulfate (pCS), diamine oxidase (DAO), trimethylamine-N-oxide (TMAO), endothelin (ET), d-lactic acid (D-LA), interferon-gamma (IFN-γ), tumor necrosis factor-alpha (TNF-α) and interleukin-6 (IL-6) in rat serum were examined follow the administration of various formulations. As shown in Fig. 7b, in model group, there was a significant decrease (*P* < 0.001) of IL-10 compared to sham group, indicating an augmented inflammation of glomerular mesangial cells [106]. Compared to the model group, the levels of IS, PCS, DAO, TMAO, ET, and D-LA significantly decreased in each administration group, with Co-Loaded Microgels group exhibiting the most notable reduction (*P* < 0.0001) (Fig. 7c–h). This reduction indicated a decrease in the circulation of intestinal urotoxins and urine toxin levels [107], highlighting the superior intestinal barrier repair capability of the Co-Loaded Microgels. Additionally, the levels of IFN-γ, TNF-α, and IL-6 were significantly decreased (*P* < 0.0001) in each administration group compared to model group (Fig. 7i–k), while the level of IL-10 showed a slight increase. Notably, consistent with staining, the Drug Loaded Microgels group and Co-Loaded Microgels group exhibited the most significant reduction in inflammation response. These findings suggest that Co-Loaded Microgels can alleviate inflammation associated with renal fibrosis.

### Regulatory effect of microgels on the gut microbiota

**Fig. 8:**
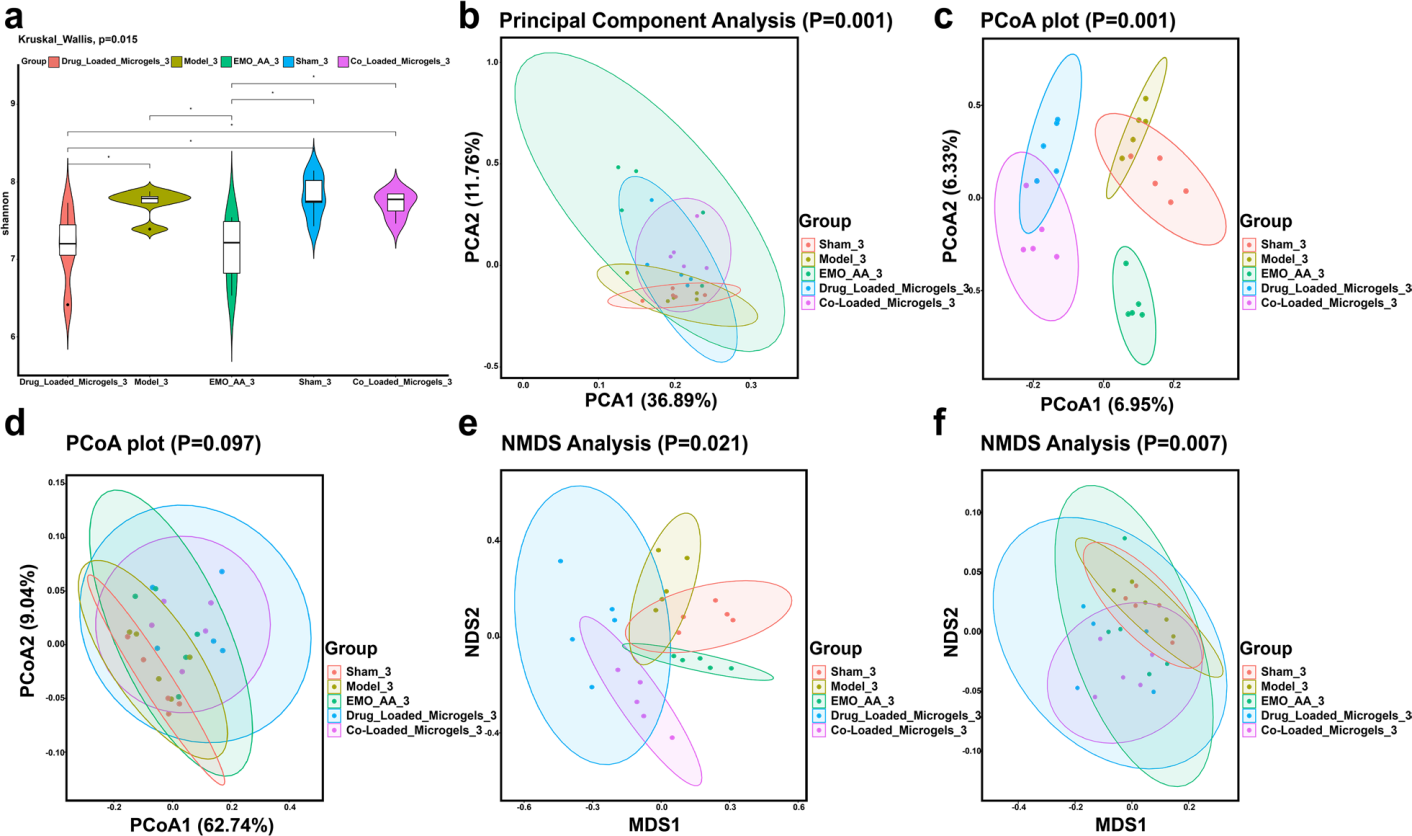
The Co-Loaded Microgels altered the gut microbiota diversity in UUO rats. **a** α-diversity of gut microbiota in terms of shannon index. **b** Principal Component analysis of weighted UniFrac distance based on 16S rDNA profiling (OTUs level) of gut microbiota in each group. **c**,** d** PCoA plot analysis of jaccrad, weighted_unifrac distance based on 16S rDNA profiling (OTUs level) in each group. **e**,** f** NMDS plot analysis of jaccrad, weighted_unifrac distance based on 16S rDNA profiling (OTUs level) in each group. (n = 5, *P < 0.05, **P < 0.01)

The regulation effect of microgels on gut microbiota was further evaluated by analyzing 16S rDNA amplicon sequencing of rats’ feces. After quality control, a total of 3,394,473 sequence reads were obtained, with an average of 69,572 reads per sample (range 400–500 bp). The α-diversity analysis showed that there was significant change between model and sham groups. Significantly, the Co-Loaded Microgels augmented the richness of the gut microflora, attributing to the presence of *L. casei* Zhang (Fig. 8a). The principal co-ordinates analysis (PCoA) and nonmetric multidimensional scaling (NMDS) plots further revealed that the bacterial community was significantly changed in model group, as evidenced by the distinct clustering of data points and the clear separation from those of sham group. In contrast, the Co-Loaded Microgels shifted the composition and structure of the bacterial community close to those of sham group, due to the prolonged intestinal retention of *L. casei* Zhang carried by microgels (Fig. 8b–f).

**Fig. 9:**
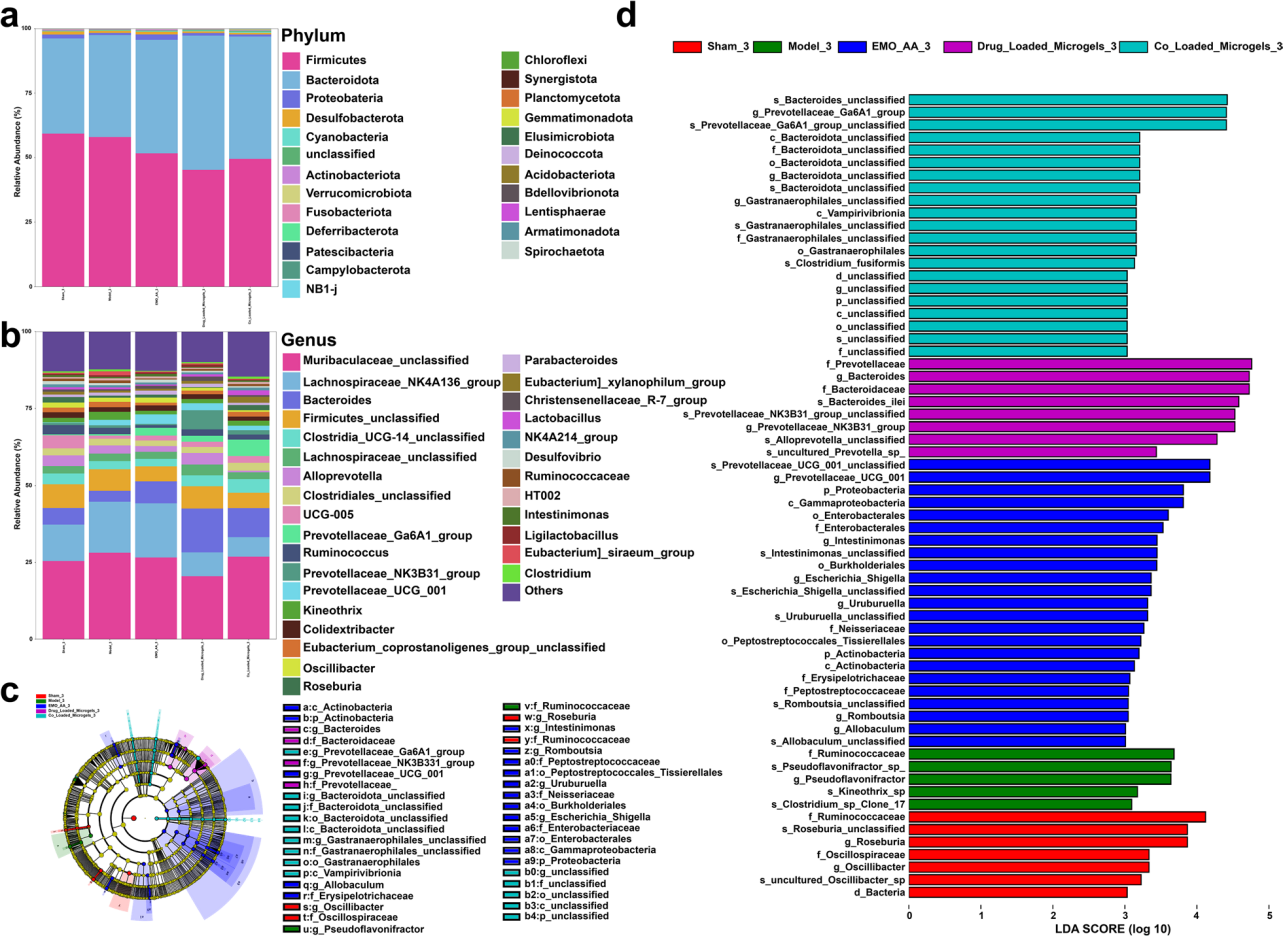
The Co-Loaded Microgels altered bacterial taxa in UUO rats. **a** Taxonomic distributions of bacteria at the phylum level (Wilcoxon rank-sum test). **b** Taxonomic distributions of bacteria at the genus level (Wilcoxon rank-sum test). **c**,** d** LefSe analysis cladogram representing the significantly different taxas among different groups (LDA score > 2.0, n = 5)

Additionally, the differential abundance of bacterial taxa among different groups was also investigated from the phylum to genus level. At the phylum level, the most abundant bacteria in all groups were *Firmicutes*, *Bacteroidetes*, *Proteobacteria*, *Desulfobacterota* and *Cyanobacteria* (Fig. 9a). In contrast to sham group, the model group exhibited a notable reduction in the abundance of *Bacteroidetes*. Remarkably, the ratio of *Firmicutes*/*Bacteroidetes* (F/B) was significantly decreased in the Co-Loaded Microgels group, in which there was a significantly increase in the abundance of *Bacteroidetes.* As is known, the ratio of F/B plays an important role in maintaining intestinal homeostasis and the higher F/B ratio is regarded as dysbiosis, which was commonly observed in CKD patients [108]. The lower F/B ratio would result in reduced accumulation of TMAO, IS and pCS, which ultimately improved the kidney functions [109]. At the genus level, as shown in Fig. 9b, *Muribaculaceae_unclassified, Lachnospiraceae_NK4A136_group, Bacteroidia,* and *Firmicutes_unclassified* were the predominant genera in all groups. In model group, it was observed that the relative abundance of *Bacteroides* was lower than sham group, which was effectively upregulated by the administration of Co-Loaded Microgels.

Further, linear discriminant analysis effect size (LEfSe) analysis was employed to identify taxa that exhibited significantly distinct abundances within each group. As shown in Fig. 9c, d, a total of 64 biomarkers were identified for gut microbiota LEfSe analysis in each group. In sham group, The most prominent bacteria were *f_Ruminococcacaea*, *g_Roseburia*, and *f_Oscillospiraceae*. Compared with sham group, the abundance of *f_Ruminococcacaea* in model group decreased significantly. Additionally, *g_Pseudoflavonifractor_sp* and *s_Clostridium_sp_Clone_17* were observed in model group, which are closely related to the progression of various kidney diseases [109]. Notably, abundances of genus *Bacteroides_unclassified* and *Bacteroidota_unclassified* were elevated in Co-Loaded Microgels group compared to model group, which correlated with the inhibition of inflammation in different organs by producing polysaccharide A (PSA) and SCFAs [110]. Overall, due to the intestinal retention of *L. casei* Zhang, the Co-Loaded Microgels were able to restore the dysbiosis of gut microbiota of UUO rats, which contributed to the amelioration of renal fibrosis through the gut-kidney axis.

## Conclusions

In this study, we have effectively presented an integrated microgel system that combined NPs@YCWPs (NPYs) and *L. casei* Zhang, using a single drug delivery system. The microgels exhibited exceptional properties, including pH-responsiveness, swelling, controlled drug release and prolonged intestinal retention of *L. casei* Zhang. More importantly, NPYs demonstrated increased uptake by RAW 264.7 cells compared to NPs alone, leading to enhanced accumulation in UUO rat’s kidney. Our in vivo animal experiments provided compelling evidence that oral administration of microgels effectively ameliorates renal fibrosis by regulating the TGF-β/Smad signaling pathway, facilitated by the hitchhiking delivery of emodin (EMO) and asiatic acid (AA), and positive modulation of the gut microbiota.

The findings in the present study could both provide a novel and highly promising therapeutic strategy for the treatment of renal fibrosis, as well as advance our understanding of chronic kidney disease through the modulation of gut flora, paving the way for more effective treatment strategies. However, there are some limitations to our study. For one thing, whether the effect of *L. casei* Zhang is dependent on the original intestinal flora needs to be clarified. And for another, given that we found that PSA and SCFA-producing bacteria, such as *Bacteroides_unclassified* and *Bacteroidota_unclassified*, were elevated in Co-Loaded Microgels group compared to model group. These results highlight the need to explore the potential mechanisms behind these beneficial effects.

### Supplementary Information

Supplementary materials 1.

## Data Availability

The datasets used and/or analysed during the current study are available from the corresponding author on reasonable request.

## Abbreviations

α-SMA: Alpha-smooth muscle actin
AA: Asiatic acid
BNPL: Benazepril
BUN: Blood urea nitrogen
CKD: Chronic kidney disease
CLSM: Confocal laser scanning microscope
CREA: Creatinine
CS: Chitosan
DAO: Diamine oxidase
DAPI: 4’,6-Diamidino-2-phenylindole
D-LA: D-lactic acid
DL: Drug loading
DMEM: Dulbecco’s modified eagle’s medium
DOX: Doxorubicin
EE: Encapsulation efficiency
EMO: Emodin
EMT: Epithelial-mesenchymal transition
ET: Endothelin
EZH 2: Enhancer of Zeste homolog 2
FAE: Follicle-associated epithelium
FBS: Fetal bovine serum
FCM: Flow cytometry
FITC: Formamide fluorescein-5-isothiocyanate
FT-IR: Fourier transform infrared spectroscopy
GIT: Gastrointestinal tract
HPLC: High-performance liquid chromatography
HYP: Hydroxyproline
IFN-γ: Interferon-gamma
IL-10: Interleukin-10
IL-6: Interleukin-6
IS: Indoxyl sulfate
IVIS: In vivo Imaging system
*L. casei* Zhang: *Lactobacillus* *casei* Zhang
MTT: Methylthiazolyldiphenyl-tetrazolium bromide
PBS: Phosphate buffered saline
pCS: P-Cresol sulfate
PSA: Polysaccharide A
PVP K30: Polyvinylpyrrolidone K30
SA: Sodium alginate
SCFAs: Short-chain fatty acids
SDS: Sodium dodecyl sulfate
SEM: Scanning electron microscope
SGF: Simulated gastric fluid
SIF: Simulated intestinal fluid
Smad 2: Recombinant mothers against decapentaplegic homolog 2
TEM: Transmission electron microscope
TGF-β1: Transforming growth factor-beta 1
TMAO: Trimethylamine-N-oxide
TNF-α: Tumour necrosis factor-alpha
UTP: Total urinary protein
UUO: Unilateral ureteral obstruction
UV: Ultraviolet
YCWPs: Yeast cell wall particles
