# Supplementary material for: Integrated oral microgel system ameliorates renal fibrosis by hitchhiking co-delivery and targeted gut flora modulation
Source: J Nanobiotechnology. 2024 Jun 1;22:305. doi: 10.1186/s12951-024-02586-2 (PMC11143587; doi:10.1186/s12951-024-02586-2)
Supplement: Supplementary file 1 — Supplementary materials 1. [file 12951_2024_2586_MOESM1_ESM.docx]

**Supplementary material**

**Integrated oral microgel system ameliorates renal fibrosis by hitchhiking co-delivery and targeted gut flora modulation**

Yu Hou^1#^, Lin Zhu^1#^, Xiaofeng Ye^1^, Qiaoying Ke^1^, Qibin Zhang^1^, Xiaowei Xie^1^, Ji-gang Piao^1*^, Yinghui Wei^1*^

1. School of Pharmaceutical Sciences, Zhejiang Chinese Medical University, Hangzhou, 311402, China

^#^Yu Hou and Lin Zhu contributed equally to this work.

^*^Correspondence: Ji-gang Piao

jgpiao@zcmu.edu.cn

Yinghui Wei

[yhw_nn@zcmu.edu.cn](mailto:yhw_nn@zcmu.edu.cn)

**
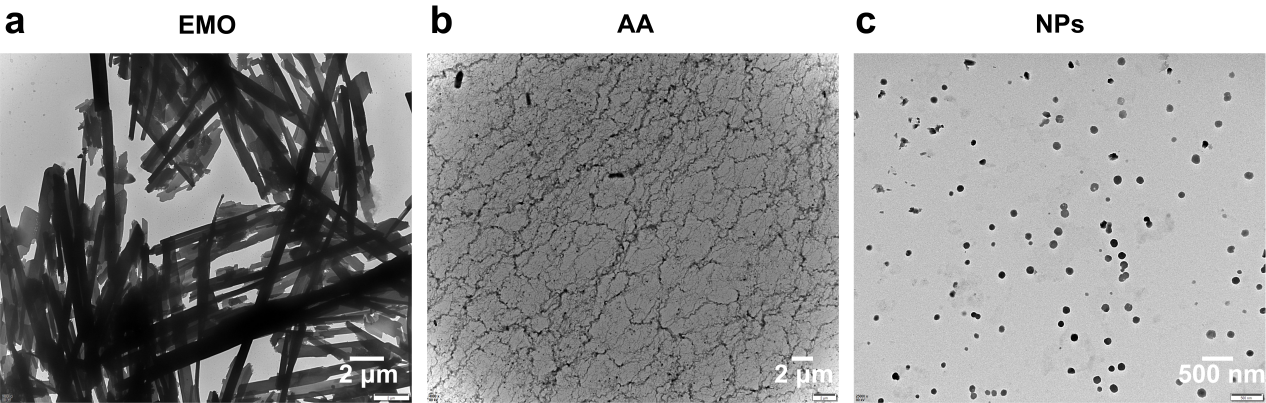
**

**Figure S1.** Representative TEM micrographs. (**a**) EMO. (**b**) AA. (**c**) NPs.


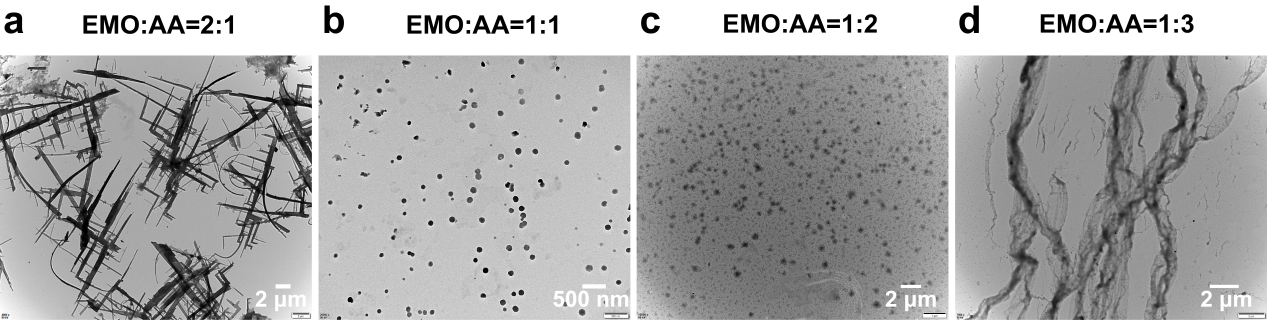


**Figure S2.** Representative TEM micrographs of NPs with different ratios of EMO to AA. (**a**) 2:1. (**b**) 1:1. (**c**) 1:2. (**d**) 1:3.


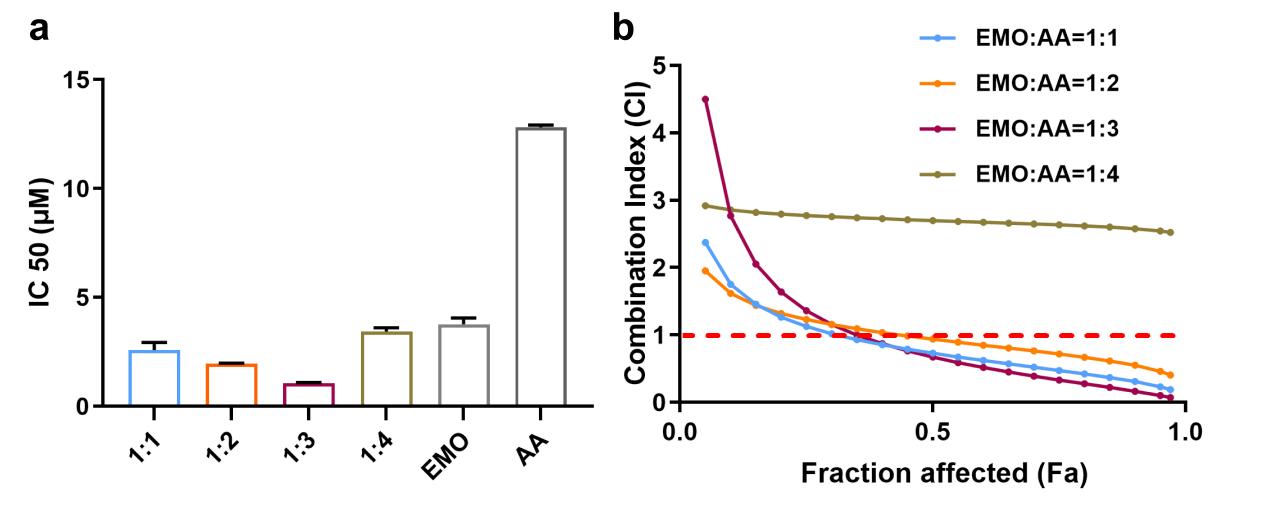


**Figure S3.** The inhibitory effect of EMO combined with AA at different molar ratios on the proliferation of HK-2 cells. (**a**) IC_50_. (**b**) CI.


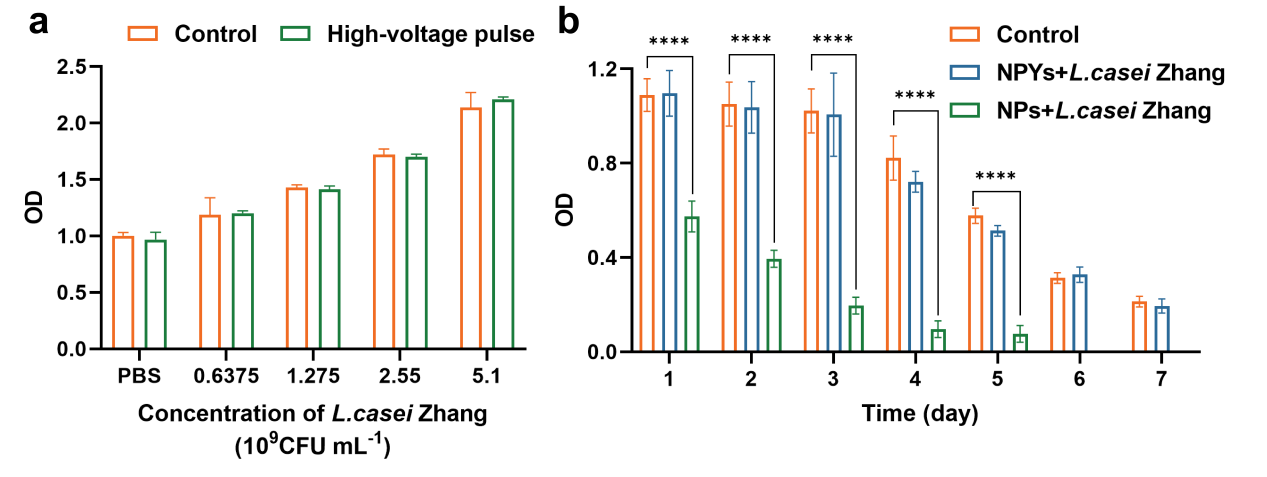


**Figure S4.** The viability of *L. casei Zhang.* (**a**) Growth curves of *L. casei* Zhang under rated voltage of or not (n = 3). (**b**) Growth curves of *L. casei* Zhang when treated with NPs and NPYs (n = 6, *****P* < 0.0001).


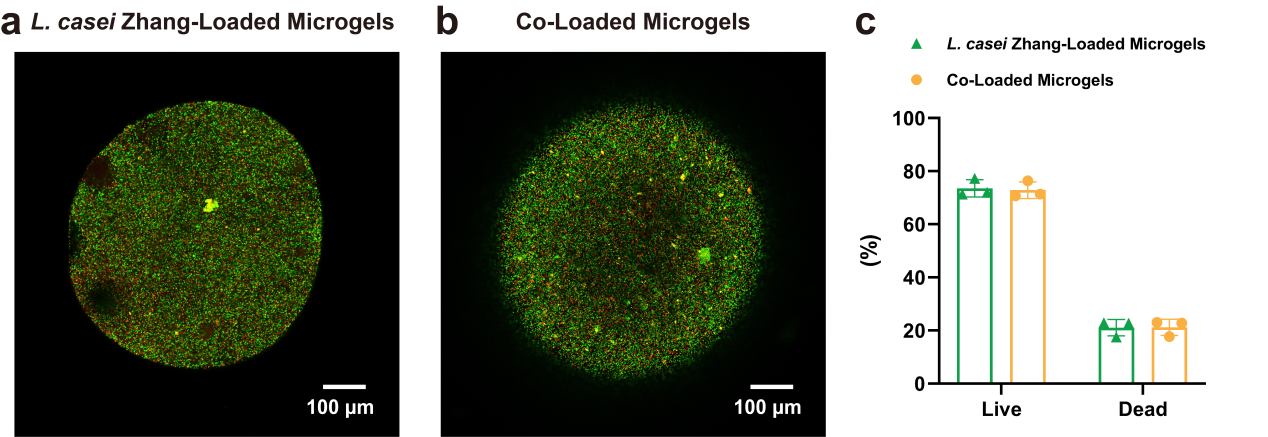


**Figure S5.** The distribution of *L. casei* Zhang within microgels. (**a**, **b**) Confocal images of live (green) and dead (red) *L. casei Zhang* within microgels. (**c**) The ratio of live and dead *L. casei* Zhang within microgels detected by FCM (n = 3).
